# Supplementary material for: USP22 controls necroptosis by regulating receptor‐interacting protein kinase 3 ubiquitination
Source: EMBO Rep. 2020 Dec 28;22(2):e50163. doi: 10.15252/embr.202050163 (PMC7857539; doi:10.15252/embr.202050163)
Supplement: Supplementary file 1 — Appendix [file EMBR-22-e50163-s001.pdf]

Appendix Table of Contents

Appendix Figure S1: CRISPR/Cas9-mediated knockout of USP22 in HT-29 cells does not affect TB-induced apoptotic cell death……………….………………..……………..………..P1

Appendix Figure S2: HT-29 USP22 KO (2g) cells display a comparable number of cells re-expressing USP22 PAM, USP22 PAM C185S or USP22 PAM C185A………………..P2

Appendix Figure S3: CRISPR/Cas9-mediated knockout of USP22 in NB4 cells does not affect TB-induced apoptotic cell death…………………………………………………..…P3

Appendix Figure S4: CRISPR/Cas9-mediated knockout of USP22 does not alter TBZ-induced cell death in MEFs and the macrophage cell lines Raw264.7 or J774A.1……………………………………………………………………………..…………P4

Appendix figure S5: HeLa cells ectopically expressing RIPK3 K-to-R mutants display a homogeneous number of RIPK3-expressing cells after Dox treatment ……………..……P5

Appendix Figure S6: siRNA mediated knockdown of USP22 in RIPK3 K518R- or RIPK3 3xKR-reconstituted HeLa cells does not affect sensitivity to TBZ-induced necroptotic cell death ………………..……………………………………………………….………..…P6

Appendix figure legends………………………………………………….………………P7

Appendix Figure S1

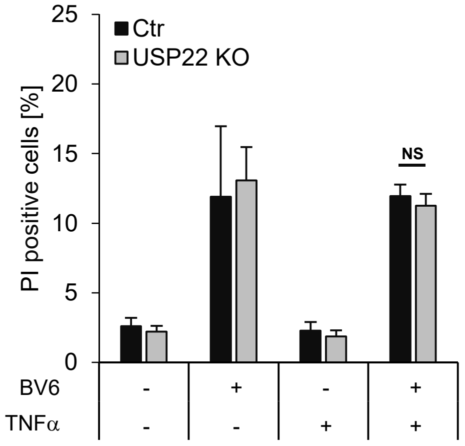

Appendix Figure S2

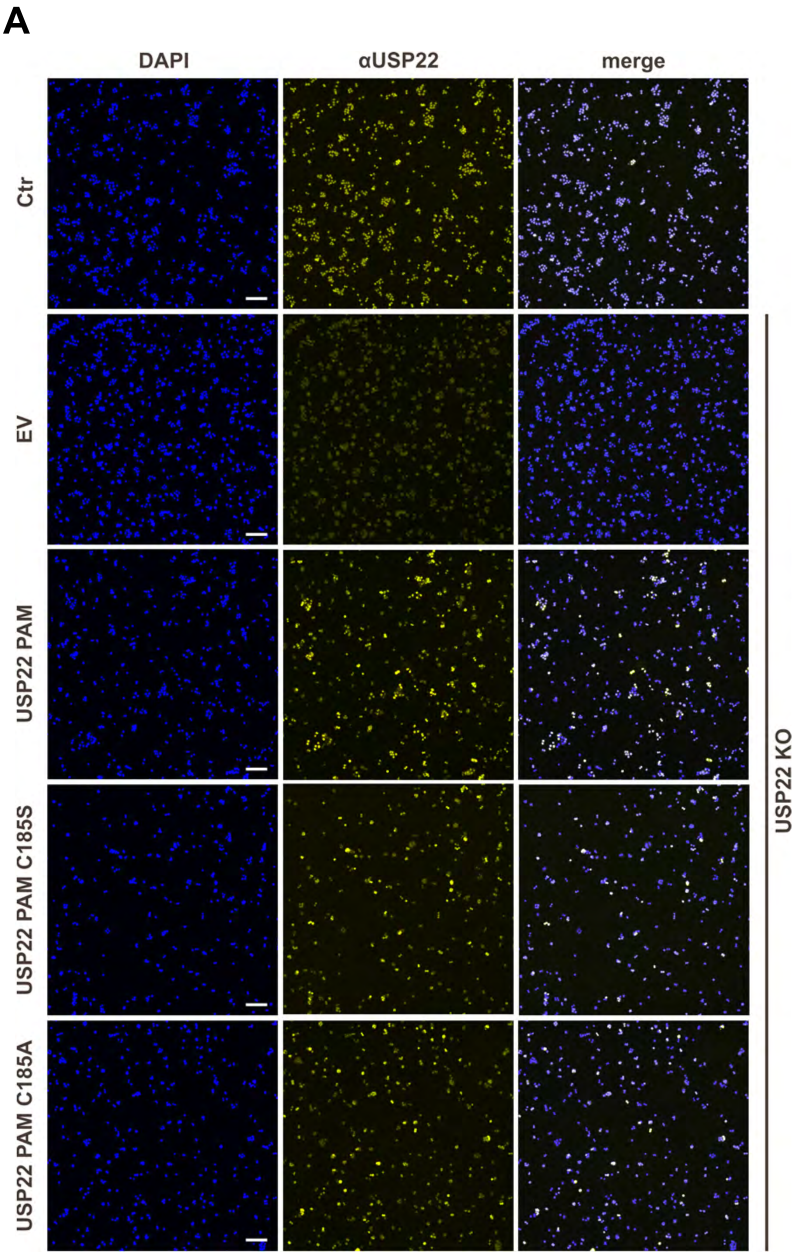

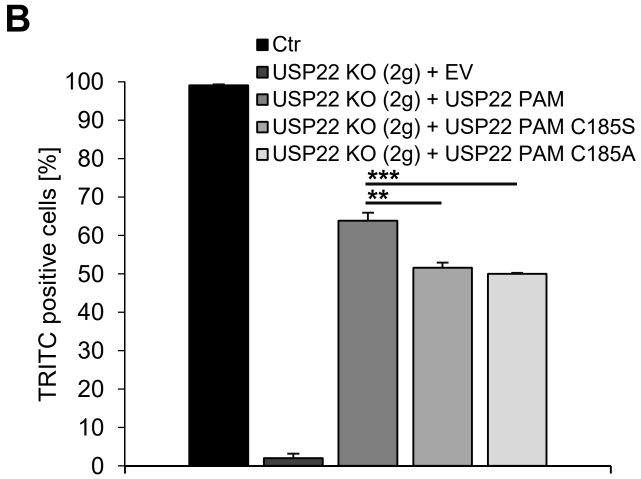

Appendix Figure S3

A

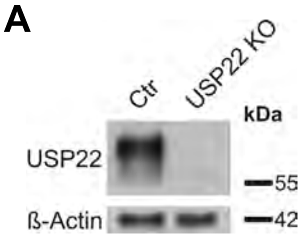

B

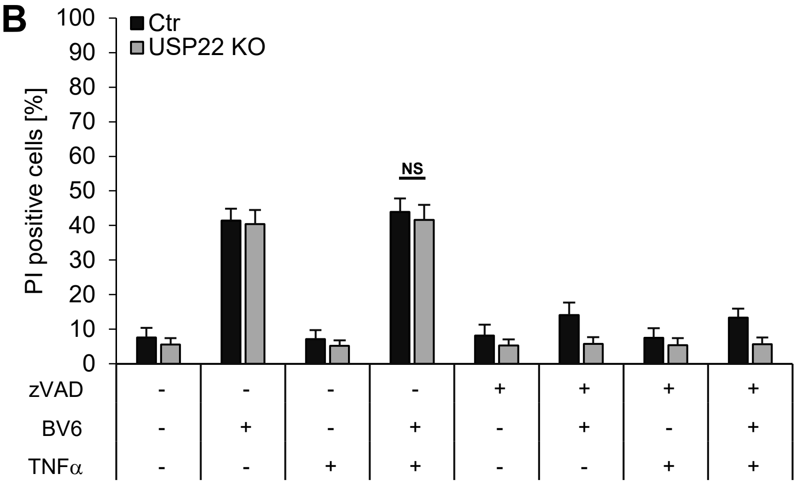

Appendix Figure S4

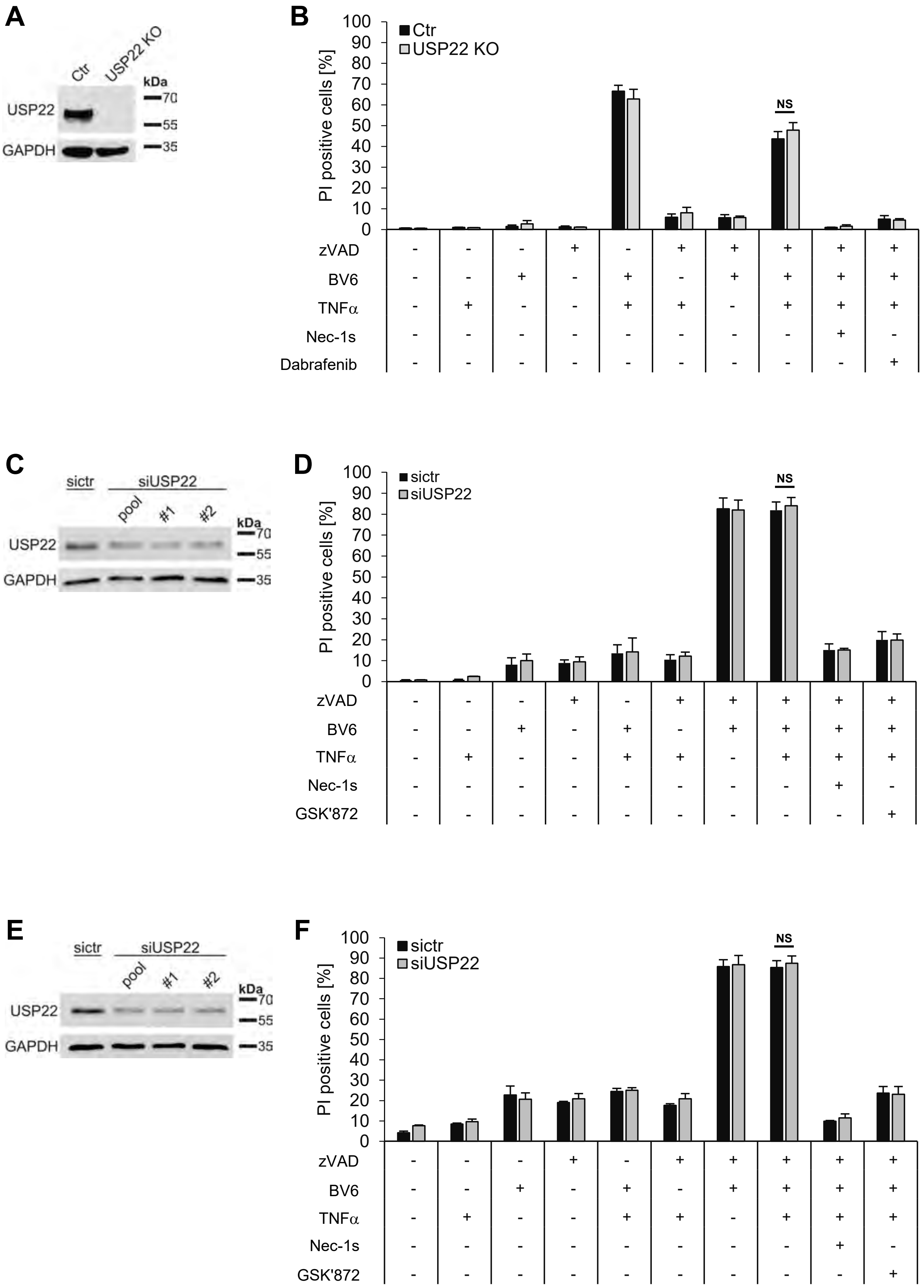

Appendix Figure S5

A

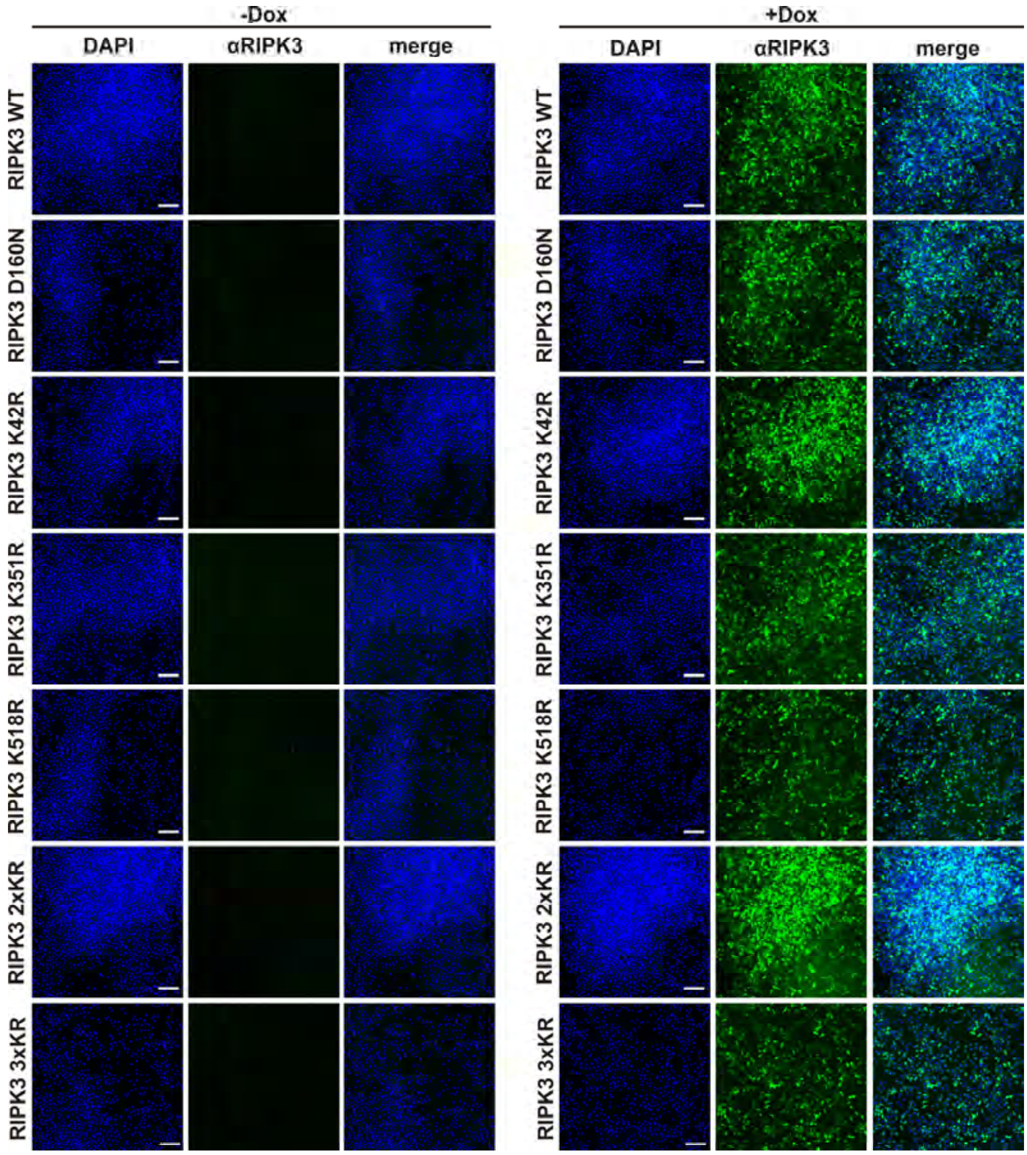

B

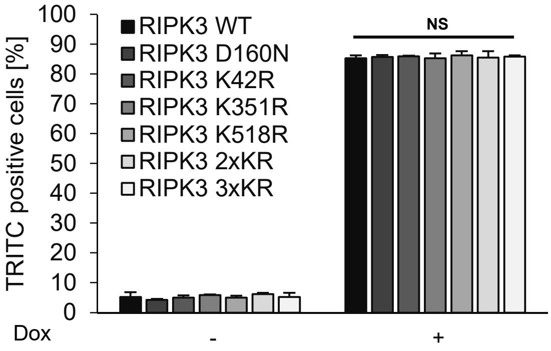

Appendix Figure S6

A

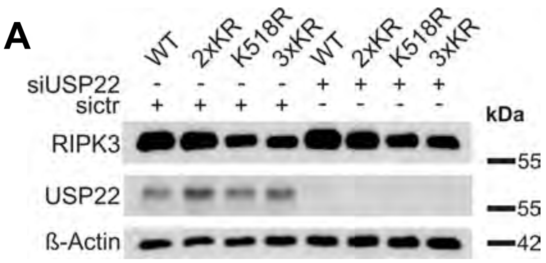

B

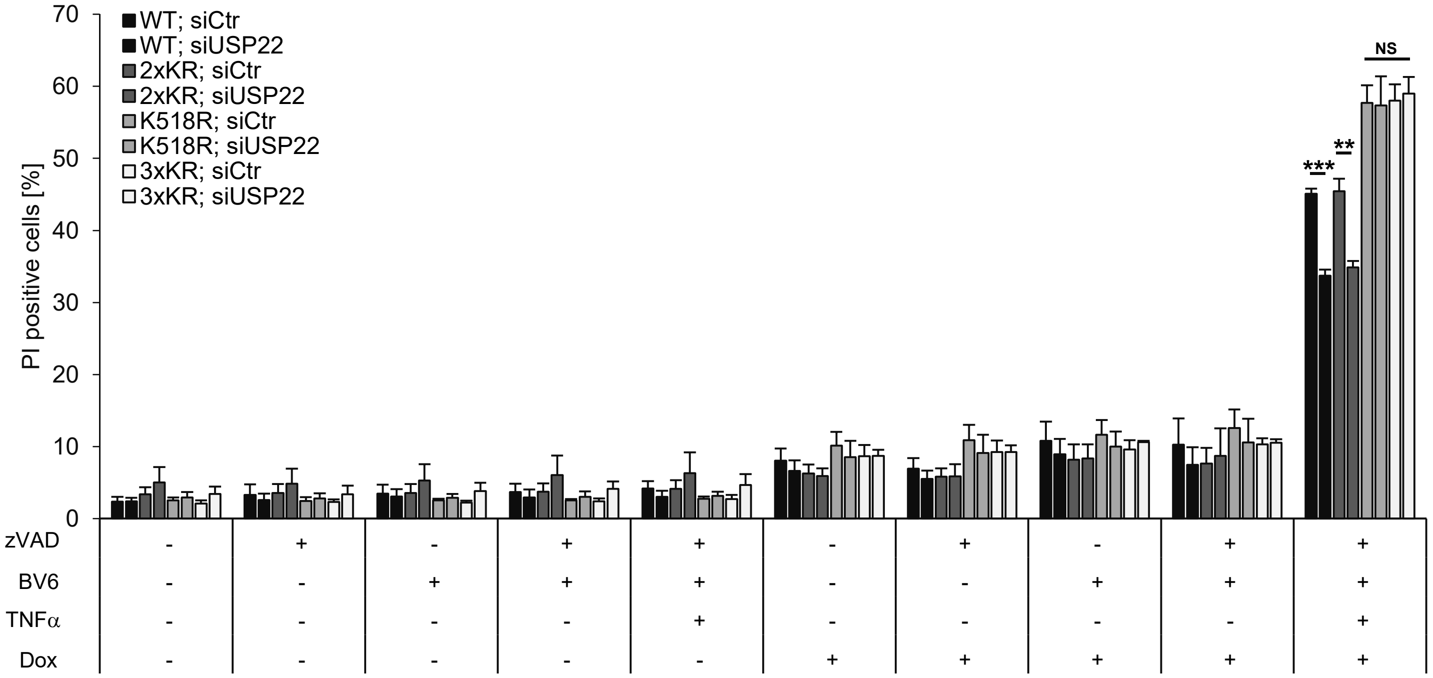

Appendix Figure S1: CRISPR/Cas9-mediated knockout of USP22 in HT-29 cells does not affect TB-induced apoptotic cell death

HT-29 CRISPR/Cas9 control (Ctr) and USP22 KO cells were treated for 72 h, as indicated, with 0.5 µM BV6 and 1 ng/ml TNFα. Cell death was determined by analysis of PI-positive nuclei.

Data information: Data represent mean ± SD; NS: not significant, by unpaired 2-tailed Student's t-test. Three independent experiments are shown.

Appendix Figure S2: HT-29 USP22 KO (2g) cells display a comparable number of cells re-expressing USP22 PAM, USP22 PAM C185S or USP22 PAM C185A

A USP22 expression was quantified using anti-USP22 immunofluorescence staining in HT-29 CRISPR/Cas9 control (Ctr) cells and in USP22 KO cells re-expressing empty vector (EV), FLAG-HA-USP22 PAM (USP22 PAM) or the corresponding mutants C185S and C185A. Scale bar represents 100 µm.

B Quantification of the percentage of TRITC-positive HT-29 cells after anti-USP22 immunofluorescence staining.

Data information: Data represent mean ± SD; ***P < 0.001; **P < 0.01, by unpaired 2-tailed Student's t-test. Three independent experiments are shown.

Appendix Figure S3: CRISPR/Cas9-mediated knockout of USP22 in NB4 cells does not affect TB-induced apoptotic cell death

A NB4 CRISPR/Cas9 control (Ctr) and USP22 KO cells were analyzed for USP22 expression by Western blotting. β-Actin served as loading control.

B NB4 control and USP22 KO cells were stimulated with 20 μM zVAD.fmk, 1 μM BV6 and 10 ng/ml TNFα. Cell death was measured after 48 h by analysis of PI-positive nuclei.

Data information: Data represent mean ± SD; NS: not significant, by unpaired 2-tailed Student's t-test. Three independent experiments are shown.

Appendix Figure S4: CRISPR/Cas9-mediated knockout of USP22 does not alter TBZ-induced cell death in MEFs and the macrophage cell lines Raw264.7 or J774A.1

A MEF CRISPR/Cas9 control (Ctr) and USP22 KO cells were analyzed by Western blotting for USP22 expression. GAPDH served as loading control.

B MEF control and USP22 KO cells were pre-treated with 10 µM Nec-1s and 20 µM Dabrafenib for 1 h. Cells were stimulated with 20 µM zVAD, 5 µM BV6 and 10 ng/ml TNFα for 24h. Cell death was determined by analysis of PI-positive nuclei.

C Raw264.7 CRISPR/Cas9 control (Ctr) and USP22 KO cells were transfected with non-silencing control siRNA (sictr) or siRNAs against USP22 (siUSP22) for 48 h at 20 nM. USP22 expression was monitored by Western blotting. GAPDH served as loading control.

D Raw264.7 control and USP22 KO cells were transfected with non-silencing control siRNA (sictr) or pooled siRNAs against USP22 (siUSP22) for 48 h at 20 nM. Cells were pre-treated with 10 µM Nec-1s and 20 µM GSK'872 for 1 h before being stimulated with 20 µM zVAD, 5 µM BV6 and 10 ng/ml TNFα for 24h. The percentage of PI-positive cells was assessed by fluorescence-based PI staining.

E J774A.1 CRISPR/Cas9 control (Ctr) and USP22 KO cells were transfected with non-silencing control siRNA (sictr) or siRNAs against USP22 (siUSP22) for 48 h at 20 nM. USP22 expression was monitored by Western blotting. GAPDH served as loading control.

F J774A.1 control and USP22 KO cells were transfected with non-silencing control siRNA (sictr) or pooled siRNAs against USP22 (siUSP22) for 48 h at 20 nM. Cells were pre-treated with 10 µM Nec-1s and 20 µM GSK'872 for 1 h prior to stimulation

with 20 µM zVAD, 5 µM BV6 and 10 ng/ml TNFα for 24h. The percentage of PI-positive cells was assessed by fluorescence-based PI staining.

Data information: Data represent mean ± SD; NS: not significant, by unpaired 2-tailed Student's t-test. Three independent experiments are shown.

Appendix figure S5: HeLa cells ectopically expressing RIPK3 K-to-R mutants display a homogeneous number of RIPK3-expressing cells after Dox treatment

A HeLa cells expressing Dox-inducible RIPK3 WT, D160N, K42R, K351R, K518R, 2xKR and 3xKR were treated with 1 µg/ml Dox overnight. RIPK3 expression was imaged using FITC-labeled anti-RIPK3 immunofluorescence staining. Scale bars represent 250 µm.

B Quantification of the percentage of FITC-positive cells treated with 1 µg/ml Dox overnight after anti-RIPK3 immunofluorescence staining.

Data information: Data represent mean ± SD; NS: not significant, by unpaired 2-tailed Student's t-test. Three independent experiments are shown.

Appendix Figure S6: siRNA mediated knockdown of USP22 in RIPK3 K518R- or RIPK3 3xKR-reconstituted HeLa cells does not affect sensitivity to TBZ-induced necroptotic cell death

A HeLa cells expressing Dox-inducible RIPK3 WT, K518R, 2xKR or 3xKR were transfected with non-silencing control siRNA (sictr) or siRNAs against USP22 (siUSP22) for 48 h at 20 nM and treated with 1 µg/ml Dox. RIPK3 and USP22 expression was analyzed by Western blotting. β-Actin served as a loading control.

B HeLa cells expressing Dox-inducible RIPK3 WT, K518R, 2xKR or 3xKR were transfected with non-silencing control siRNA (sictr) or pooled siRNAs against USP22 (siUSP22) for 48 h at 20 nM and treated with 1 µg/ml Dox, as indicated. Cells were pre-treated with 20 µM zVAD.fmk and 5 µM BV6 for 1 h after 10 ng/ml TNFα was added for 4 h. The percentage of PI-positive cells was assessed by fluorescence-based PI staining.

Data information: Data represent mean ± SD; **P < 0.01; ***P < 0.001; NS: not significant, by unpaired 2-tailed Student's t-test. Three independent experiments are shown.
