# Supplementary material for: USP22 controls necroptosis by regulating receptor‐interacting protein kinase 3 ubiquitination
Source: EMBO Rep. 2020 Dec 28;22(2):e50163. doi: 10.15252/embr.202050163 (PMC7857539; doi:10.15252/embr.202050163)
Supplement: Supplementary file 2 — Expanded View Figures PDF [file EMBR-22-e50163-s002.pdf]

Expanded View Figures

Figure EV1. Loss of USP22 in HT-29 cells does not affect TB-induced apoptosis or NF-κB activation upstream of IκBα.

A Whole cell lysates of HT-29 parental, CRISPR/Cas9 control (ctr), and USP22 KO cells were analyzed by Western blotting for the indicated proteins. β-Actin was used as a loading control.
B HT-29 control and USP22 KO cells were treated with 20 μM zVAD.fmk, 0.5 μM BV6, and 1 ng/ml TNFα for 6 h, and cell death was determined by analysis of PI-positive nuclei.
C HT-29 control and USP22 KO cells were stimulated with 20 μM zVAD.fmk, 0.5 μM BV6, and 1 ng/ml TNFα for 18 h. Cells were additionally treated with 30 μM Nec-1s, 20 μM GSK'872, 20 μM Dab, and 10 μM NSA, as indicated. Cell death was determined by analysis of PI-positive nuclei.
D HT-29 control and USP22 KO cells were stimulated with 10 ng/ml TNFα for 5 and 15 min. Protein expression of IκBα, phosphorylated IκBα, and USP22 were examined by Western blotting. Vinculin was used as a loading control.
E HT-29 control and USP22 KO cells were treated for 48 h, as indicated, with 0.5 μM BV6 and 1 ng/ml TNFα. Cell death was determined by analysis of PI-positive nuclei.
F HT-29 control cells and USP22 KO cells, generated with three (USP22 KO #1) or with two (USP22 KO (2g)) USP22 gRNAs, were stimulated with 20 μM zVAD.fmk, 0.5 μM BV6, 1 ng/ml TNFα, and 30 μM Nec-1s for 18 h. The percentage of PI-positive cells was assessed by fluorescence-based PI staining.
G Jurkat CRISPR/Cas9 control (Ctr) control and USP22 KO cells were analyzed for USP22 expression by Western blotting. GAPDH served as loading control.
H Jurkat control and USP22 KO cells were pre-treated with 10 μM Nec-1s before stimulation with 1 μM BV6 and 10 ng/ml TNFα. Cell death was measured after 18 h by analysis of PI-positive nuclei.
I Jurkat control and USP22 KO cells were pre-treated with 10 μM Nec-1s or 20 μM Dabrafenib before stimulation with 1 μM BV6 and 10 ng/ml TNFα for 8 h. Cell death was determined by analysis of PI-positive nuclei.

Data information: Data represent mean ± SD; *$P < 0.05$; **$P < 0.01$; ***$P < 0.001$, NS: not significant, by unpaired 2-tailed Student's *t*-test. Three independent experiments performed in triplicate are shown.

 © 2021 The Authors

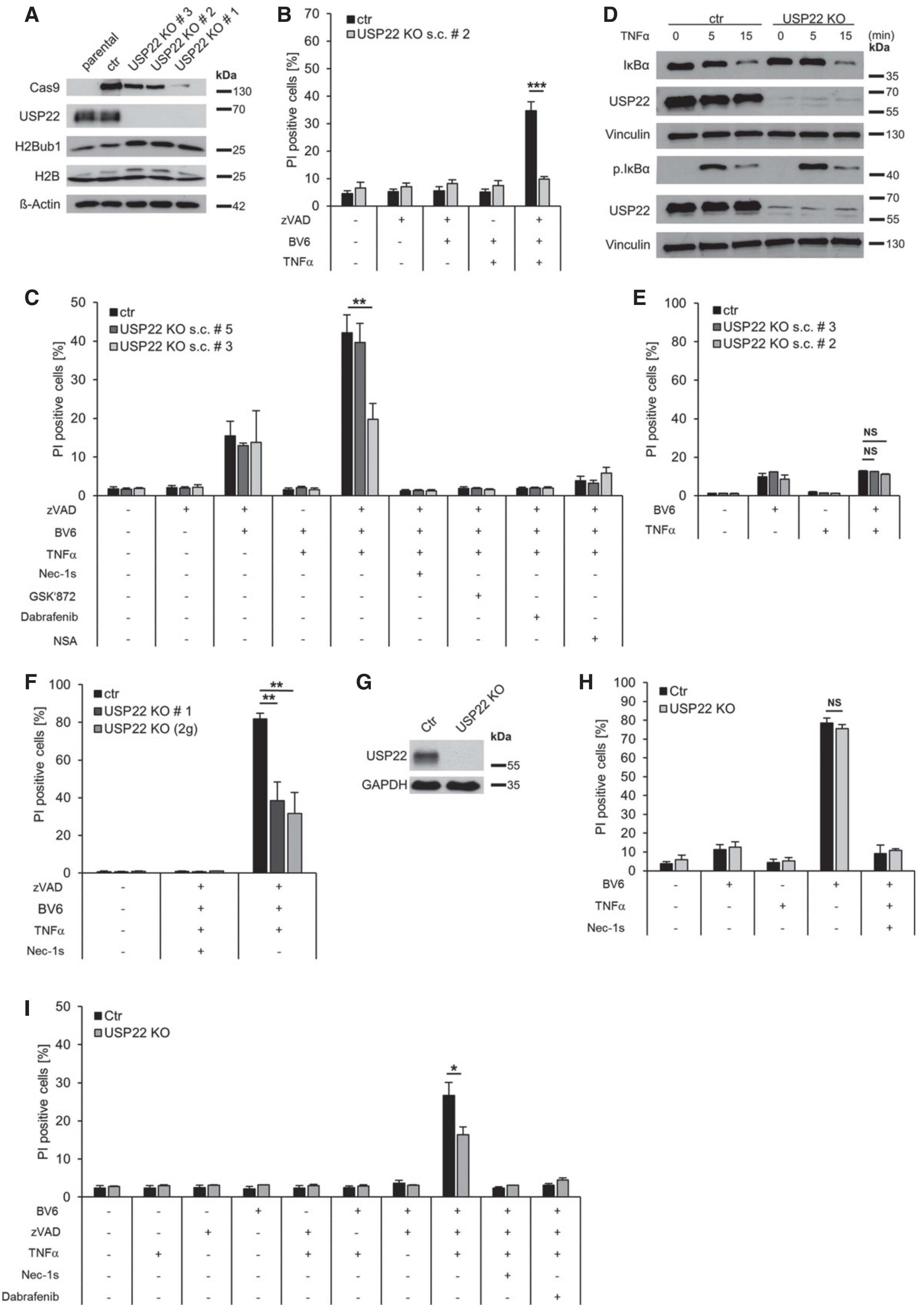
A
parental
ctr
USP22 KO # 3
USP22 KO # 2
USP22 KO # 1
Cas9
USP22
H2Bub1
H2B
ß-Actin
kDa
130
70
25
25
42
B
ctr
USP22 KO s.c. # 2
PI positive cells [%]
***
zVAD
BV6
TNFα
C
ctr
USP22 KO s.c. # 5
USP22 KO s.c. # 3
PI positive cells [%]
**
zVAD
BV6
TNFα
Nec-1s
GSK'872
Dabrafenib
NSA
D
ctr
USP22 KO
TNFα
(min)
kDa
IκBα
USP22
Vinculin
p.IκBα
USP22
Vinculin
E
ctr
USP22 KO s.c. # 3
USP22 KO s.c. # 2
PI positive cells [%]
NS
NS
BV6
TNFα
F
ctr
USP22 KO # 1
USP22 KO (2g)
PI positive cells [%]
**
**
zVAD
BV6
TNFα
Nec-1s
G
Ctr
USP22 KO
kDa
USP22
GAPDH
55
35
H
Ctr
USP22 KO
PI positive cells [%]
NS
BV6
TNFα
Nec-1s
I
Ctr
USP22 KO
PI positive cells [%]
*
BV6
zVAD
TNFα
Nec-1s
Dabrafenib

Figure EV1.

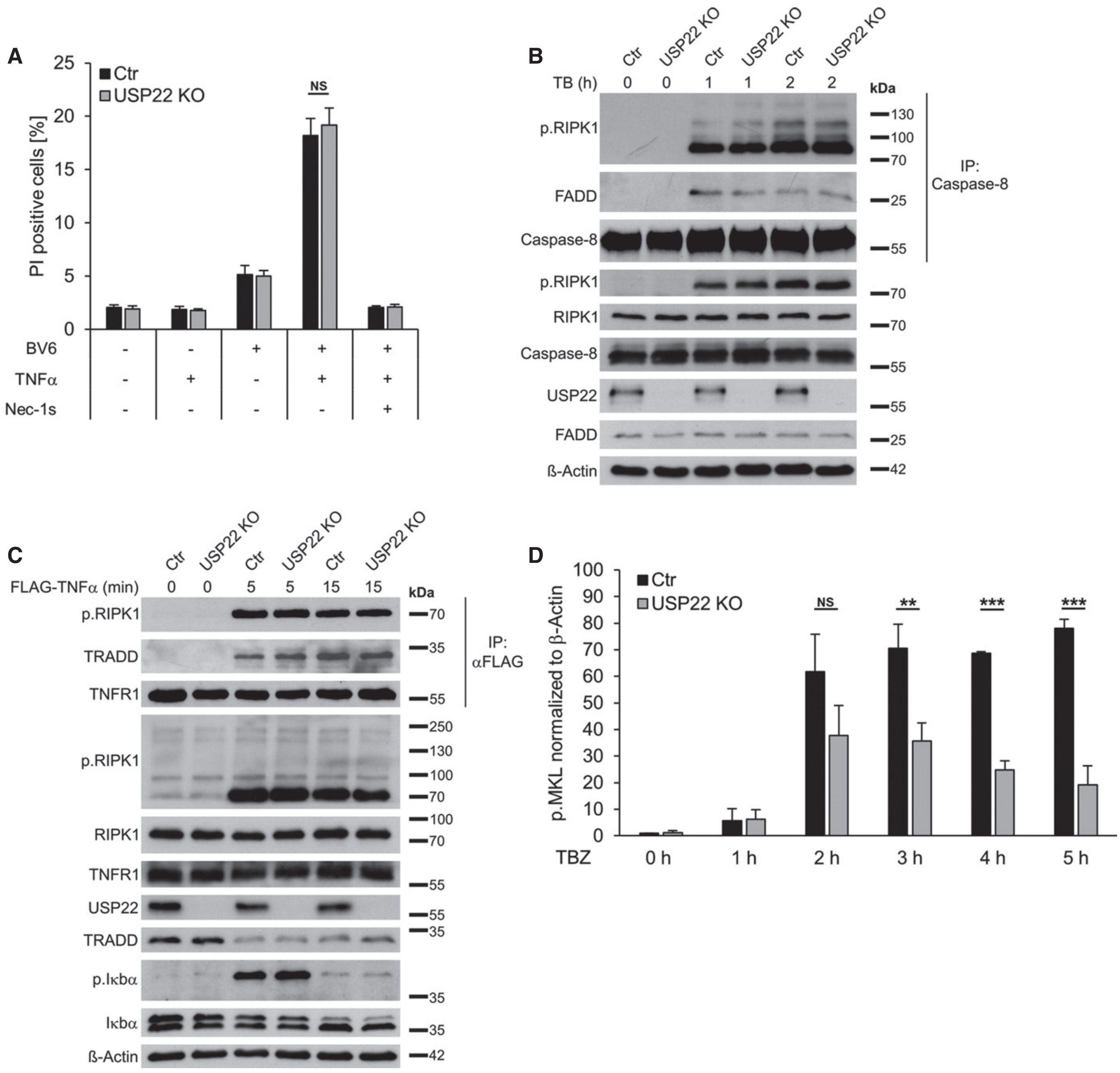

Figure EV2. USP22 KO in HeLa TRex RIPK3 does not affect TB-induced apoptosis or complex I and II formation.

A HeLa TRex RIPK3 CRISPR/Cas9 control (Ctr) and USP22 KO cells were treated with 5 μM BV6 and 10 ng/ml TNFα for 24 h. Cell death was determined by analysis of PI-positive nuclei.

B HeLa TRex RIPK3 CRISPR/Cas9 control (Ctr) and USP22 KO cells were pre-treated with 5 μM BV6 for 1 h before being stimulated with 10 ng/ml TNFα for 1 and 2 h, after which caspase-8 was immunoprecipitated, followed by analysis by Western blotting with the indicated antibodies. β-Actin served as loading control.

C HeLa TRex RIPK3 CRISPR/Cas9 control (Ctr) and USP22 KO cells were starved in serum-free DMEM for 2 h. Cells were stimulated with 1 μg/ml FLAG-hTNF for 5 and 15 min as indicated in the Materials and Methods section. FLAG-hTNF was immunoprecipitated and analyzed by Western blotting with the indicated antibodies. β-Actin served as loading control.

D RIPK3 expression in HeLa TRex CRISPR/Cas9 control and USP22 KO cells was induced by Dox treatment. For quantification, phosphorylated MLKL levels of both cell lines were first normalized to β-Actin levels followed by normalization to the 0 h time point of HeLa TRex CRISPR/Cas9 control cells.

Data information: Data represent mean ± SD; $^{**}P < 0.01$; $^{***}P < 0.001$; NS: not significant, by unpaired 2-tailed Student's *t*-test. Three independent experiments performed in triplicate are shown. In panel D, quantification of blots from three independent experiments.

 © 2021 The Authors

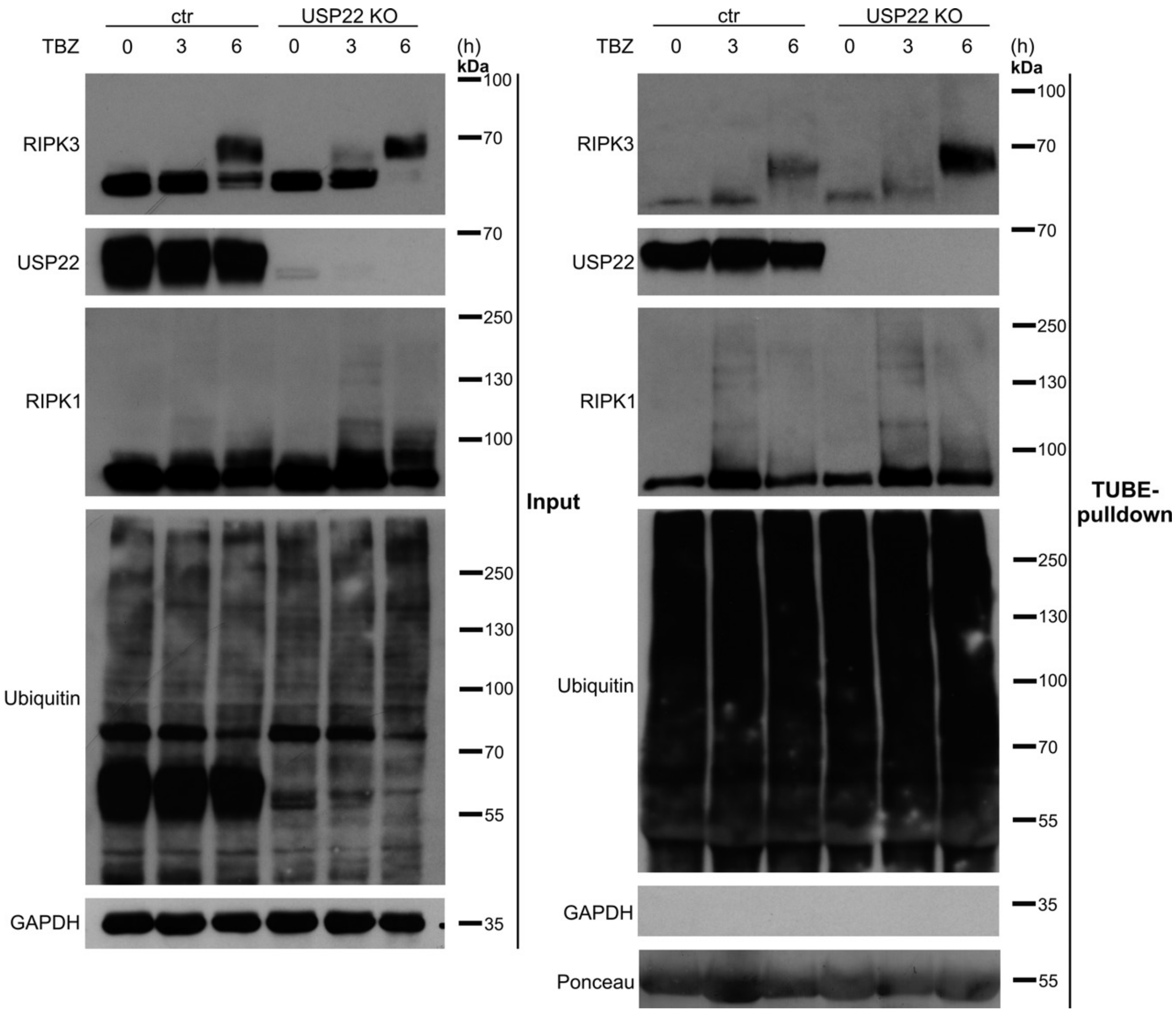

Figure EV3. USP22 KO in HT-29 cells does not alter TBZ-induced RIPK1 ubiquitination.

HT-29 control and USP22 KO cells were stimulated with 20 μM zVAD.fmk, 0.5 μM BV6, and 1 ng/ml TNFα for 3 and 6 h. Ubiquitinated proteins were enriched by GST-TUBE pull-down. Protein expression of RIPK1, RIPK3, USP22, and ubiquitinated RIPK1, RIPK3, and USP22 was monitored using Western blotting with the indicated antibodies. GAPDH served as a loading control. Ponceau staining was used to confirm equal loading of GST-TUBE.

Figure EV4. Ubiquitin remnant profiling identifies RIPK3 K518 as USP22-dependent ubiquitin target during necroptosis.

A RIPK3 ubiquitin sites detected by ubiquitin remnant profiling upon USP22 KO and TBZ treatment. The mean of two biological replicates is depicted. Only RIPK3 sites are shown that were identified in both replicates.

B Fragment spectrum of TBZ-treated RIPK3 di-glycine-modified peptide corresponding to K518. The b- and y-ions detected are highlighted.

C SILAC-labeled HT-29 CRISPR/Cas9 control (ctr) and USP22 KO cells were stimulated with 20 μM zVAD.fmk, 0.5 μM BV6, and 1 ng/ml TNFα for 6 h. The percentage of PI-positive cells was assessed by fluorescence-based PI staining.

D SILAC-labeled HT-29 control and USP22 KO cells were treated with 20 μM zVAD.fmk, 0.5 μM BV6, and 1 ng/ml TNFα for 6 h and analyzed by Western blotting with the indicated antibodies. GAPDH served as a loading control. The arrow marks specific phosphorylated MLKL bands.

E HeLa cells expressing Dox-inducible RIPK3 WT or 3xKR were incubated with 30 μM Nec-1s or 20 μM Dab and 1 μg/ml Dox overnight, as indicated. Cells were stimulated with 20 μM zVAD.fmk, 0.5 μM BV6, and 1 ng/ml TNFα for 2 h. 100 μg of each lysate was incubated with 400 U/μl λ-phosphatase for 30 min at 30°C. RIPK3 protein expression was monitored by Western blotting. β-Actin was used as loading control.

F HeLa cells expressing Dox-inducible RIPK3 WT or K518R were incubated with 1 μg/ml Dox overnight. Cells were pre-treated with 20 μM zVAD.fmk and 5 μM BV6 for 1 h. After pre-treatment, 10 ng/ml TNFα was added for 2 h. Levels of inducible RIPK3 expression were analyzed by Western blotting under reducing and non-reducing conditions. β-Actin served as loading control. The asterisk marks putative RIPK3 dimers.

Data information: Data represent mean ± SD; $^{**}P < 0.01$, by unpaired 2-tailed Student's *t*-test. Three independent experiments are shown.

© 2021 The Authors

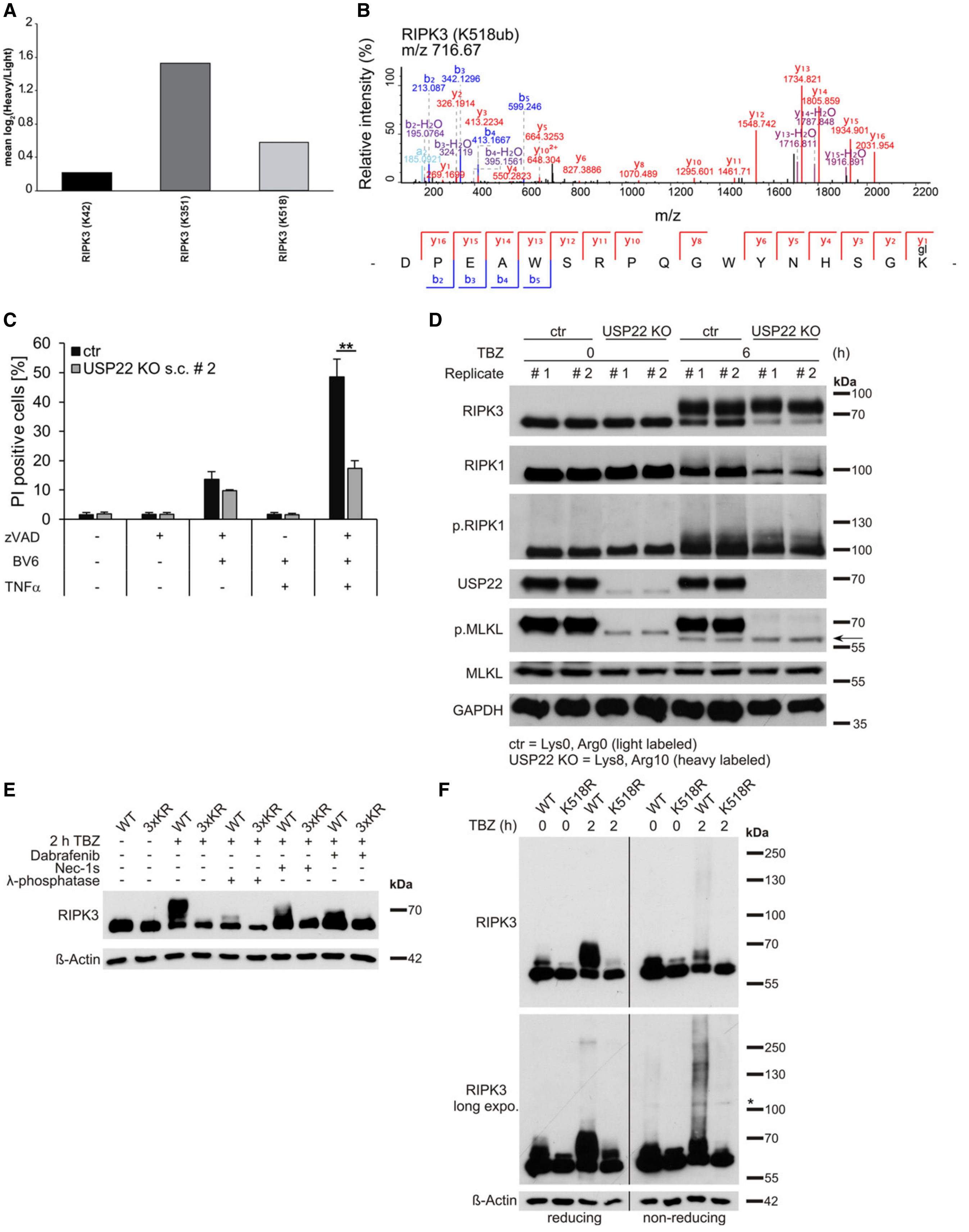
A
mean log2(Heavy/Light)
RIPK3 (K42)
RIPK3 (K351)
RIPK3 (K518)
B
RIPK3 (K518ub)
m/z 716.67
Relative intensity (%)
m/z
C
ctr
USP22 KO s.c. # 2
PI positive cells [%]
zVAD
BV6
TNFα
D
ctr USP22 KO ctr USP22 KO
TBZ 0 6 (h)
Replicate
RIPK3
RIPK1
p.RIPK1
USP22
p.MLKL
MLKL
GAPDH
kDa
ctr = Lys0, Arg0 (light labeled)
USP22 KO = Lys8, Arg10 (heavy labeled)
E
2 h TBZ
Dabrafenib
Nec-1s
λ-phosphatase
RIPK3
ß-Actin
F
TBZ (h)
RIPK3
RIPK3 long expo.
ß-Actin
reducing
non-reducing

Figure EV4.

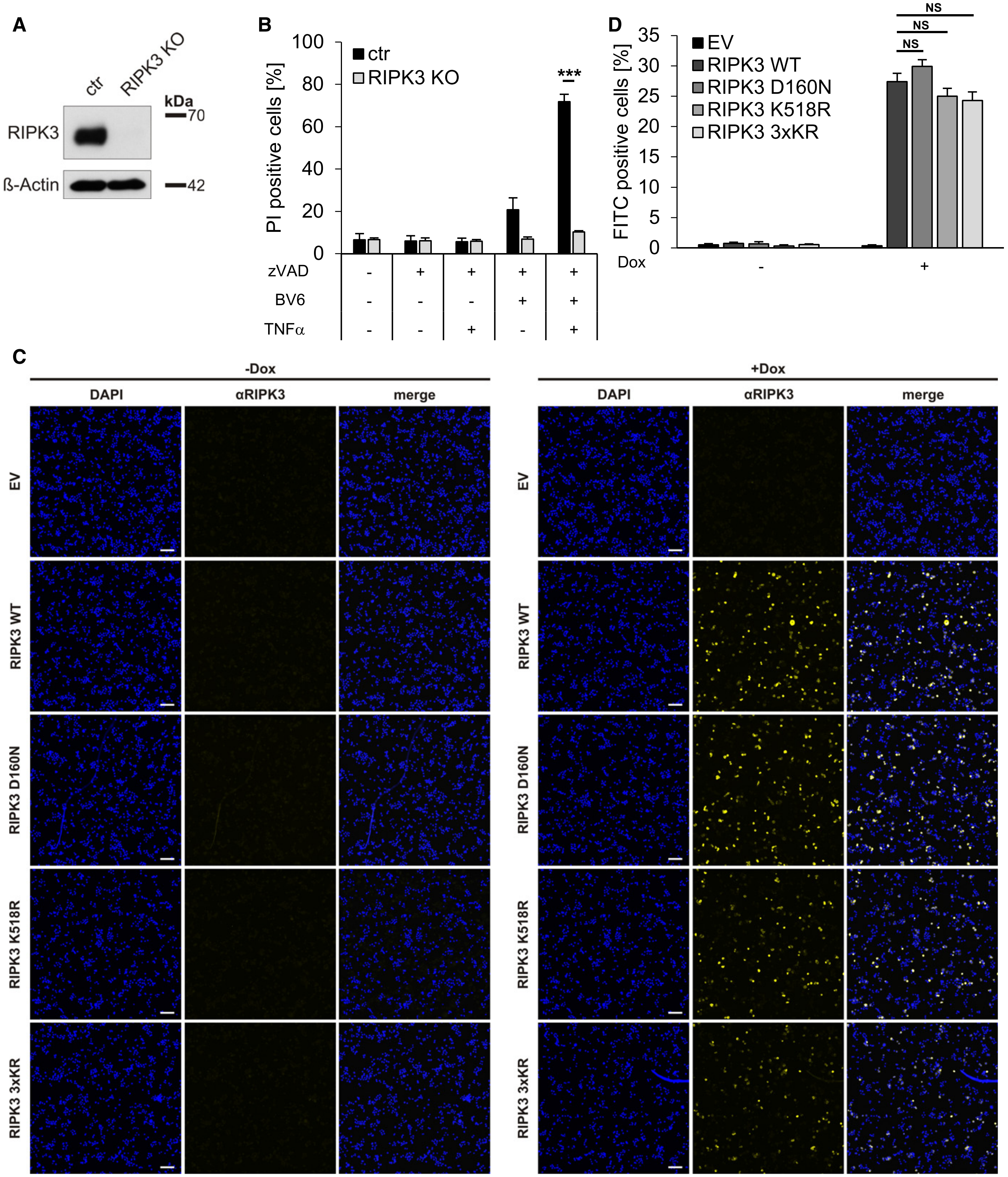

Figure EV5.

 © 2021 The Authors

Figure EV5. HT-29 RIPK3 KO cells, reconstituted with RIPK3 mutants, show homogenous RIPK3 expression upon Dox treatment.

A HT-29 CRISPR/Cas9 control (ctr) and RIPK3 KO cells were analyzed by Western blotting for RIPK3 expression. β-Actin served as loading control.

B HT-29 control and RIPK3 KO cells were stimulated with 20 μM zVAD.fmk, 0.5 μM BV6, and 1 ng/ml TNFα for 18 h. The percentage of PI-positive cells was assessed by fluorescence-based PI staining.

C HT-29 cells expressing EV and Dox-inducible RIPK3 WT, D160N, K518R, and 3xKR were treated with 1 μg/ml Dox overnight. Strep-RIPK3 expression was imaged using anti-RIPK3 immunofluorescence staining. Scale bars represent 100 μm.

D Quantification of FITC-positive cells after RIPK3 immunofluorescence staining of HT-29 cells expressing Dox-inducible RIPK3 WT, D160N, K518R, and 3xKR.

Data information: Data represent mean ± SD; ***$P < 0.001$, NS: not significant, by unpaired 2-tailed Student's *t*-test. Three independent experiments are shown.

© 2021 The Authors
